# Breaking Down the Stereotypes of Science by Recruiting Young Scientists

**DOI:** 10.1371/journal.pbio.0020279

**Published:** 2004-10-12

**Authors:** Jamie Schaefer, Steven A Farber

## Abstract

Thomas Jefferson University Science Outreach Program brings the scientific method into the classroom

If you ask the average ten year old in America what a scientist looks like, they almost always describe an older man with crazy white hair and a lab coat. If you ask a group of adolescents how many have looked through a microscope, few raise their hands. If you discuss the implications of genetic research with a group of high school students, they're likely to cut your next class. The reason why these students have such profound stereotypes of scientists and are less than enthusiastic about science's impact on society is simple—the lack of exposure they receive during their pre-college education. According to a preliminary study conducted at Leicester University in England, students are often repeatedly confronted with stereotypes of science and scientists via television, cartoon, and comic book characters as well as uninformed adults or peers (McDuffie 2001).

A university set in a major city has the resources to change the mindset of urban students and engage them in the exciting field of science. At Thomas Jefferson University (TJU), located in Philadelphia, Pennsylvania, a team of scientists and educators has developed a program that breaks down the stereotypes of the science field and allows students to engage in real, live experiments at their own schools.

## Why Are Such Programs Necessary?

Around the world, educators face difficult choices in focusing educational goals with limited resources. In the United States, the *No Child Left Behind Act*, which guides school funding policy, currently places an emphasis on literacy and math in schools, with the result that best programs and practices in education are increasingly directed toward these two areas. Unfortunately, science education has become a lesser priority. Teachers are not given adequate resources to allow students to “get their hands dirty” during science lessons. If we want a society that is interested and knowledgeable about the need for scientific research, the basic principles of the life sciences need to be integrated early in the pre-college curriculum (Sylwester 2001). We developed the Thomas Jefferson University Science Outreach Program (TJUSOP) to address this key issue and provide inquiry-based educational strategies through collaborative efforts between the university's faculty and partnering school districts.

## How Are We Making a Difference?

Using the TJU facilities and laboratories, this innovative program integrates life science into the education of students between the ages of eight and eighteen from Pennsylvania, New Jersey, and Delaware. The mission of the TJUSOP is to foster an enthusiasm for science education, promote interest in future participation in biology-related fields, and allow all students the opportunity to learn life science through a hands-on, student-centered approach to instruction. The program is a supplement to the established curriculum, developed to support the content knowledge that is taught at each grade level. Teachers are invited to attend a professional development workshop held at the beginning of the school year where they receive training and resources for the units. Then, TJUSOP educators assist the teachers and students in their own classroom in running a weeklong experiment. This allows a large amount of group work to be completed simultaneously, even when teachers are faced with time constraints and large class sizes. This program is at no cost to the districts participating and is funded through the Jefferson Medical College and the Kimmel Cancer Center, as well as through the generosity of several local and national groups including Glaxo Smith Kline, the Christopher Ludwick Foundation, the Joan and Joseph Fernandez Family Foundation, the Brook J. Lenfest Foundation, the Foerderer Foundation, Drinker Biddle and Reath, and the Pennsylvania Department of Agriculture.

Since its inception in August of 2002, this program has reached over 2,000 students and 75 teachers through our one-week zebrafish classroom experiments, our hands-on zebrafish and Drosophila facility tours in conjunction with Dr. James Jaynes (a Drosophila scientist at the Kimmel Cancer Center), and our High School Mentorship program held each summer. One of the main goals of TJUSOP is to reach students from ethnic and economic groups that are underrepresented in the scientific community. We have successfully partnered with the School District of Philadelphia, where 84.9% of the students come from ethnic backgrounds other than Northern European and 80% of the students are eligible for free or reduced-cost meals. This district, among many others, receives pre- and post-instruction for all teachers and students at no cost to the district, allowing it to improve the quality of its science education. Although we target these school districts, it is important to note that the US is facing a problematic decrease in the number of Americans, of any background, entering the science and engineering workforce. According to a National Science Foundation report, “If action is not taken now to change these trends, we could reach 2020 and find that the ability of U.S. research and education institutions to regenerate has been damaged and that their preeminence has been lost to other areas of the world” (National Science Board Committee 2004). In regard to this unsettling discovery, TJUSOP welcomes all districts to participate and hopes to secure funding to double the number of students reached per year.

## Anyone Can Be a Scientist

**Figure 1:**
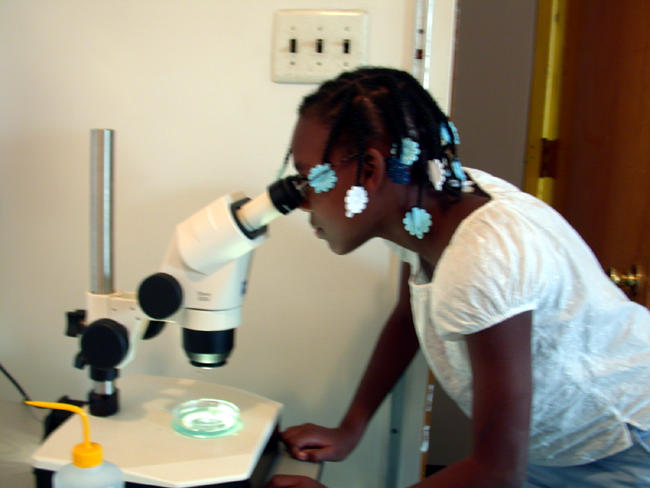
A Philadelphia Student Uses the Zeiss Stereomicroscope during the Weeklong Experiment

Our pedagogical approach to experiments allows students and teachers to become scientists, following the scientific process from beginning to end. Our live, one-week classroom experiments for the fourth, seventh, and tenth grades use zebrafish, a popular model organism for genetic research. A curriculum sample is as follows: in the seventh-grade unit, the students mate albino (recessive trait) male and a wild-type (dominant trait) female zebrafish in order to observe what the offspring will look like. Students form hypotheses, such as that the young offspring will look like the mother and the older offspring will be striped. Throughout the week, students observe and record embryos developing a head, tail, and notochord and pigment development. By the end of the experiment, a live heartbeat can be seen as well as the individual blood cells flowing throughout the larvae using a stereomicroscope TJUSOP provides (Figure 1).

**Figure 2:**
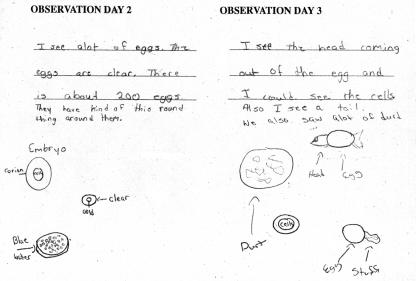
An Example of a Seventh-Grade Journal Entry

Grade-specific scientific journals are given to the students. The journals contain an introduction to TJUSOP and the experiment, background information about zebrafish in research, scientific vocabulary words used throughout the unit, and a word search activity. Students are given the title of “Junior Scientists” in grades 4 and 7 and “Student Scientists” in grade 10 and are asked to record the research question, a hypothesis, daily observations, and the conclusion of the experiment (Figure 2).

## How to Break the Stereotypes of What Science Is

TJUSOP allows student participants to use scientific tools, talk with real scientists, and gain scientific knowledge so they can become informed members of their communities. Upon asking a fourth-grade student why she thought it was important to learn about science using zebrafish and the microscope, the student wrote, “I think it is important because we can find facts about oursefs [sic].” This sounds like a good start.

For more information about the program, or if you would like to get involved in the initiative, please contact Jamie Schaefer, at jamie. E-mail: Schaefer@mail.jci.tju.edu or visit http://www.kimmelcancercenter.org/scienceoutreachprogram.

## Abbreviations

TJU: Thomas Jefferson University
TJU-SOP: Thomas Jefferson University Science Outreach Program
